# Evaluation of Antimicrobial Peptide–Antibiotic Combination Treatment for Tackling Ocular and Systemic *Staphylococcus aureus* Infections

**DOI:** 10.3390/ijms27125573

**Published:** 2026-06-20

**Authors:** Eman Khalid Barahim, Ella P. Smith, Sheau Ting Yong, Thet Tun Aung, Rajamani Lakshminarayanan, Imran Mohammed, Harminder S. Dua, Graham R. Wallace, Jose R. Hombrebueno, Saaeha Rauz, Darren S. J. Ting

**Affiliations:** 1 Academic Unit of Ophthalmology, Department of Inflammation and Ageing, School of Infection, Inflammation and Immunology, College of Medicine and Health, University of Birmingham, Birmingham B15 2TT, UK; ekb460@student.bham.ac.uk (E.K.B.); exs260@student.bham.ac.uk (E.P.S.); s.t.yong@bham.ac.uk (S.T.Y.); g.r.wallace@bham.ac.uk (G.R.W.); j.m.romero@bham.ac.uk (J.R.H.); s.rauz@bham.ac.uk (S.R.); 2 Department of Biological Sciences, College of Sciences, University of Jeddah, Jeddah 21589, Saudi Arabia; 3 Singapore Eye Research Institute, Singapore National Eye Centre, Singapore 169856, Singapore; thet.tun.aung@seri.com.sg (T.T.A.); lakshminarayanan.rajamani@seri.com.sg (R.L.); 4 Ophthalmology and Visual Sciences Academic Clinical Program (EYE-ACP), Duke-NUS Medical School, Singapore 169857, Singapore; 5 Department of Pharmacy and Pharmaceutical Sciences, National University of Singapore, Singapore 117543, Singapore; 6 Laboratory of Antimicrobial Drug Discovery, School of Optometry and Vision Sciences, College of Biomedical and Life Sciences, Cardiff University, Cardiff CF24 4HQ, UK; mohammedi5@cardiff.ac.uk; 7 Academic Ophthalmology, Mental Health and Clinical Neurosciences, School of Medicine, University of Nottingham, Nottingham NG7 2RD, UK; profdua@gmail.com; 8 Department of Ophthalmology, Queen’s Medical Centre, Nottingham NG7 2UH, UK; 9 Birmingham and Midland Eye Centre, Sandwell and West Birmingham NHS Trust, Birmingham B18 71H, UK

**Keywords:** antibiotic, antibiotic resistance, antimicrobial resistance, host defence peptide, staphylococcus

## Abstract

*Staphylococcus aureus* is a leading cause of bacterial keratitis and antimicrobial resistance-associated death globally. This study aimed to evaluate the efficacy of CaD23, a human-derived hybrid antimicrobial peptide (AMP), in combination with antibiotics in treating *S. aureus* infections. The efficacy of CaD23 and six medically important antibiotics (amikacin, cefuroxime, chloramphenicol, fosfomycin, vancomycin and levofloxacin) was examined against six strains of methicillin-sensitive and methicillin-resistant *S. aureus* using a minimum inhibitory concentration (MIC) assay. CaD23–antibiotic interactions were evaluated using checkerboard and time–kill kinetics assays. 3,3′-dipropylthiadicarbocyanine iodide (DiSC_3,5_) cytoplasmic membrane depolarisation assay was performed to examine the mechanism of action. Overall, CaD23 exhibited good efficacy against all MSSA and MRSA (MIC = 16–32 μg/mL [6.7–13.3 μM]). Of 20 peptide–antibiotic–organism combinations, 19 (95%) combinations demonstrated positive interactions, with six (31.6%) and 13 (68.4%) exhibiting synergistic (FICI = 0.293–0.412) and additive effects (FICI = 0.521–0.890), respectively. CaD23 was able to achieve complete bacterial eradication significantly faster than cefuroxime and levofloxacin (15 min vs. 8–24 h). When used at a sub-MIC concentration, CaD23 could accelerate the killing of *S. aureus* of cefuroxime from 8–24 h to within 1 h and enhance the activity of levofloxacin by 90%. CaD23 was shown to rapidly depolarise the inner membrane of *S. aureus* within seconds of the treatment. In conclusion, CaD23–antibiotic combination therapy serves as a useful strategy for tackling drug-resistant ocular and systemic *S. aureus* infections.

## 1. Introduction

Antimicrobial resistance (AMR), especially bacterial AMR, has emerged as one of the major global health threats, with around 5 million AMR-associated deaths reported annually [1,2,3]. This is likely to increase to 10 million deaths by 2050 if not addressed urgently. A number of factors have contributed to the rising challenges of AMR, including the excessive and injudicious use of antibiotics, lack of access to antibiotics, inadequate infection prevention and control, and the lack of development of new antibiotics due to dwindling pharmaceutical interest [1,3]. Importantly, there have been limited new antibiotics introduced to the market since the 1980s [4,5].

*Staphylococcus aureus*, a Gram-positive, sphere-shaped (coccus) bacterium, is a common human microbiota that is found in around 20–30% of healthy individuals. It can colonise various sites of the human body, including the nose, skin, throat, intestine, and eye/ocular surface [6,7] and is capable of causing a wide range of opportunistic infections, including infectious keratitis (IK), endophthalmitis, soft tissue infections, osteomyelitis, pneumonia, and endocarditis [6,8,9]. It is recognised as a leading cause of AMR-associated death and forms part of the distinct group of pathogens, named ESKAPEE, encompassing *Enterococcus* spp., *S. aureus*, *Klebsiella pneumonia, Acinetobacter baumannii*, *Pseudomonas aeruginosa*, *Enterobacter* spp., and *E. coli* [10]. *S. aureus* are notoriously known to cause drug-resistant hospital-acquired infections [1,3,11]. They can acquire AMR genes effectively via multiple mechanisms, such as altered drug targets and drug accessibility, increased efflux pump, and enzymatic drug inactivation [12], consequently reducing the effectiveness of available antibiotic treatments and resulting in treatment failure [6,13]. Recent studies have shown that methicillin-resistant *S. aureus* (MRSA) infections are continuing to rise in many regions (e.g., around 60% hospital-acquired *S. aureus* infections were multidrug-resistant), posing significant therapeutic challenges [1,11,14,15].

Within the context of ocular infections, *S. aureus* is a common causative organism for IK, which is the leading cause of corneal blindness and vision impairment globally [16]. It was estimated that 8–36% of bacterial keratitis is attributed to *S. aureus*, and it increases to 24–46% when coagulase-negative *Staphylococci* species are included [8,16,17]. It has been reported that MRSA causes around one-third of *S. aureus* keratitis in some regions, with a high proportion of them being multidrug-resistant [14,18,19]. Broad-spectrum topical antibiotics are currently the primary treatment for bacterial keratitis. However, the efficacy of antibiotics is being increasingly challenged by a number of factors, including rising AMR [14,19,20], polymicrobial infections [21,22], and lack of new antibiotics. Consequently, this can lead to treatment failure and serious ocular complications, such as corneal melt, perforation, and endophthalmitis [23,24]. All these issues highlight an urgent need for novel antimicrobial therapies and strategies.

Antimicrobial peptides (AMPs), also known as host defence peptides (HDPs), have recently demonstrated promise as a novel antimicrobial therapy in view of their broad-spectrum and unique antimicrobial action [25,26,27,28]. Recently, we developed a novel 18-mer human-derived hybrid AMP, named CaD23, based on the rationale hybridisation of human cathelicidin (LL-37) and human beta-defensin-2 (HBD-2), with strong in vitro and in vivo antimicrobial activity against Gram-positive bacterial infections and synergistic/additive interaction with antibiotics [29,30]. Rationale hybridisation of the functional components of two different AMPs has previously shown to be an effective strategy to enhance the efficacy and safety and/or reduce the length of AMPs (thereby reducing the cost of production and increasing the chance of clinical translation) [31,32]. This was similarly demonstrated in our previous study where we showed that CaD23 was able to enhance the activity of native HBD-2 (41-mer) and maintain a similar antimicrobial activity as LL-37 (37-mer) but with a much shorter peptide length (i.e., <50% of its original length) [29].

However, previous studies only focussed on limited laboratory reference strains of *S. aureus* and the interaction of CaD23 with two types of antibiotics, namely levofloxacin and amikacin. In this study, we hypothesised that CaD23 will exhibit potent antibacterial efficacy against a range of laboratory and clinical strains of drug-resistant *S. aureus* and enhance the effectiveness of a wide range of medically important antibiotics.

## 2. Results

### 2.1. In Vitro Antimicrobial Efficacy of Peptide and Antibiotics

The antibiogram of six different classes of medically important antibiotics, including amikacin, cefuroxime, chloramphenicol, fosfomycin, levofloxacin, and vancomycin, and CaD23 against six different strains of *S. aureus* is presented in Table 1. SA29213 and MRSA43300 were resistant to one class of antibiotic, USA300 was resistant to two classes of antibiotics, and BH1CC showed multidrug resistance (i.e., resistance to ≥3 classes of antibiotics). CaD23 exhibited similarly good efficacy against all six strains of MSSA and MRSA (MIC = 16–32 μg/mL [or 6.7–13.3 μM]; Table 1). CaD23 was also examined in the presence of the physiological salt concentration (150 mM NaCl), which is known to influence/affect the efficacy of peptides. The MIC of CaD23 remained largely unchanged in the presence of 150 mM NaCl (80% [4/5 strains] within 2-fold change; Table 2).

### 2.2. Checkerboard Assay

A total of 20 peptide–antibiotic–organism combinations were investigated for their potential synergistic antibacterial activity. Among all, 19 (95%) of the combinations demonstrated positive interaction, with six (31.6%) and 13 (68.4%) exhibiting synergistic and additive effects, respectively (Table 3). CaD23–amikacin combination treatment demonstrated a synergistic effect against MRSA43300 (FICI = 0.398) and MRSA-OS (FICI = 0.293), strong additive effects against SA29213 (FICI = 0.842), SH1000 (FICI = 0.521), and USA300 (FICI = 0.625), and an indifferent effect against BH1CC (FICI = 1.113). The CaD23–levofloxacin combination exhibited a synergistic effect against MRSA-OS (FICI = 0.412) and strong additive effect against all other five strains of *S. aureus* (FICI = 0.590–0.890). Further examination of the interaction between CaD23 and four other antibiotics, including cefuroxime, chloramphenicol, fosfomycin, and vancomycin, against MRSA-OS and BH1CC demonstrated either a synergistic effect (FICI = 0.250–0.406) or additive effect (FICI = 0.583–0.833; Table 3).

### 2.3. Time–Kill Kinetics Assay

**Figure 1:**
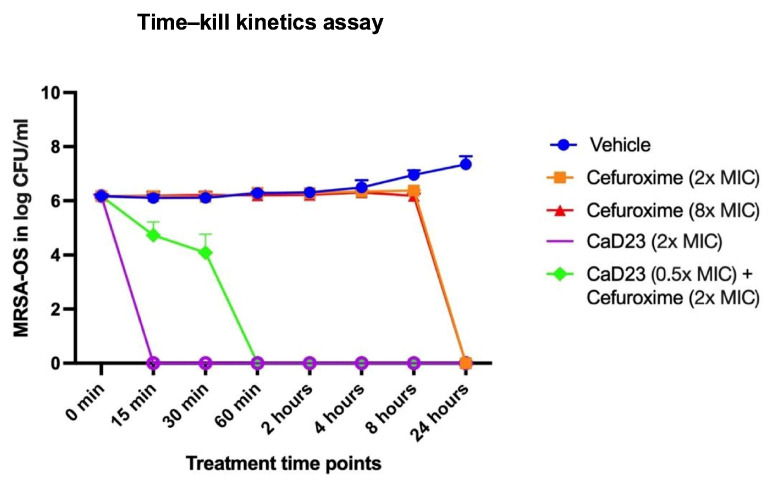
Time–kill kinetics assay demonstrating the time- and concentration-dependent antibacterial effect of CaD23 monotherapy, cefuroxime monotherapy, and CaD23–cefuroxime combination therapy against methicillin-resistant *Staphylococcus aureus* (MRSA-OS) over 24 h. MRSA-OS incubated with sterile deionised water serves as the untreated control/vehicle. “0 min” represents the starting inoculum, which is around 6 log_10_CFU/mL. Data are presented as mean ± standard deviation (depicted in error bars) of two to three independent experiments.

In view of the synergistic action of CaD23–cefuroxime combination therapy against MRSA-OS, a time–kill kinetics assay was further performed to determine the concentration- and time-dependent antimicrobial effects of CaD23 monotherapy, cefuroxime monotherapy, and CaD23–cefuroxime combination therapy against MRSA-OS over 24 h (Figure 1). When CaD23 was administered alone at a 2× MIC concentration (64 μg/mL), it achieved a complete eradication of MRSA-OS within 15 min. This was significantly faster than cefuroxime, which, at concentrations of 2× MIC (8 μg/mL), 8× MIC (32 μg/mL) and 32× MIC (128 μg/mL), was only able to achieve a complete killing of MRSA-OS after 8–24 h (i.e., CaD23 was at least 32-fold or 97% faster than cefuroxime; Figure 1). For the combined treatment, the addition of CaD23 at a sub-MIC level (16 μg/mL; 0.5× MIC) was able to significantly expedite the antimicrobial action of cefuroxime by at least eight-fold (or 87.5% faster), accelerating the complete eradication of MRSA-OS from 8–24 h to 1 h post-treatment. When administered alone, CaD23 at a sub-MIC value (16 μg/mL; 0.5× MIC) was not able to achieve a complete killing of MRSA-OS.

**Figure 2:**
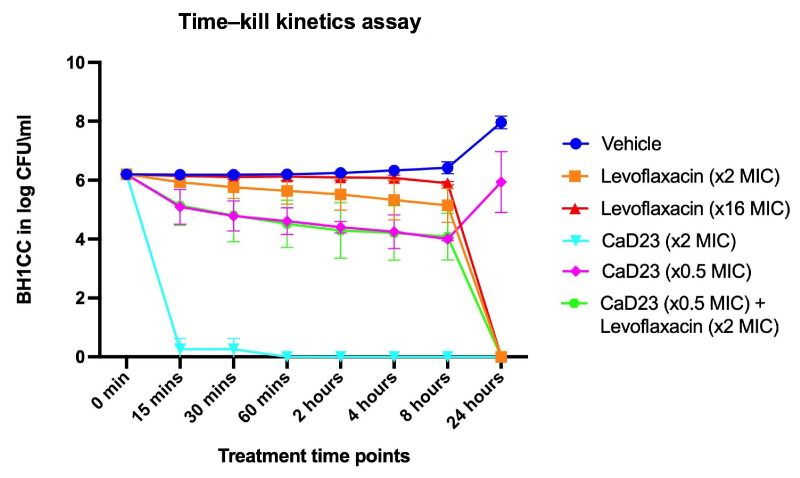
Time–kill kinetics assay demonstrating the time- and concentration-dependent antibacterial effect of CaD23 monotherapy, levofloxacin monotherapy, and CaD23–levofloxacin combination therapy against methicillin-resistant *Staphylococcus aureus* (BH1CC) over 24 h. BH1CC incubated with sterile deionised water serves as the untreated control/vehicle. “0 min” represents the starting inoculum, which is around 6 log_10_CFU/mL. Data are presented as mean ± standard deviation (depicted in error bars) of two to three independent experiments.

To examine the generalisability of the above findings, we evaluated the potential synergistic effect between CaD23 and another class of antibiotic (levofloxacin) against another clinical strain of *S. aureus* (BH1CC) using a time–kill kinetics assay (Figure 2). CaD23 monotherapy (2× MIC, 64 μg/mL) was able to achieve complete bacterial eradication within 15 min, whereas levofloxacin monotherapy, at either a 2× MIC (8 μg/mL) or 16× MIC (64 μg/mL) concentration, was only able to achieve 100% killing at 8–24 h post-treatment (Figure 2), which is 32–96 times slower than CaD23. This also suggests that the antimicrobial action of levofloxacin is more time-dependent than concentration-dependent. The addition of CaD23 (0.5× MIC; 16 μg/mL) to levofloxacin (2× MIC; 8 μg/mL) did not expedite the action of levofloxacin, but it enhanced the activity by 90% (1 logCFU/mL reduction in bacteria when compared to levofloxacin alone [2× MIC]).

### 2.4. Cytoplasmic Membrane Depolarisation Assay (DiSC_3,5_)

**Figure 3:**
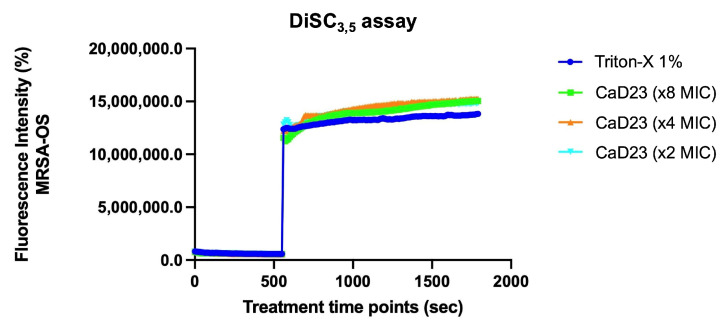
The DiSC_3,5_ assay showing rapid cytoplasmic/inner membrane depolarisation effect of CaD23 against an ocular clinical isolate of methicillin-resistant *Staphylococcus aureus* (MRSA-OS) signal within seconds of the addition of CaD23 at around 600 s (where the fluorescein signal was stable).

The mechanism of action of CaD23 against MRSA-OS was investigated using the DiSC_3,5_ cytoplasmic membrane depolarisation assay. CaD23 (at 2×, 4× an 8× MIC concentrations) was shown to rapidly depolarise the inner membrane of MRSA-OS within seconds of the addition of treatment (Figure 3). This finding corresponded with the time–kill kinetics assays where CaD23 was shown to achieve rapid killing of the bacteria within 15 min.

### 2.5. CCK-8 Cell Viability Assay

**Figure 4:**
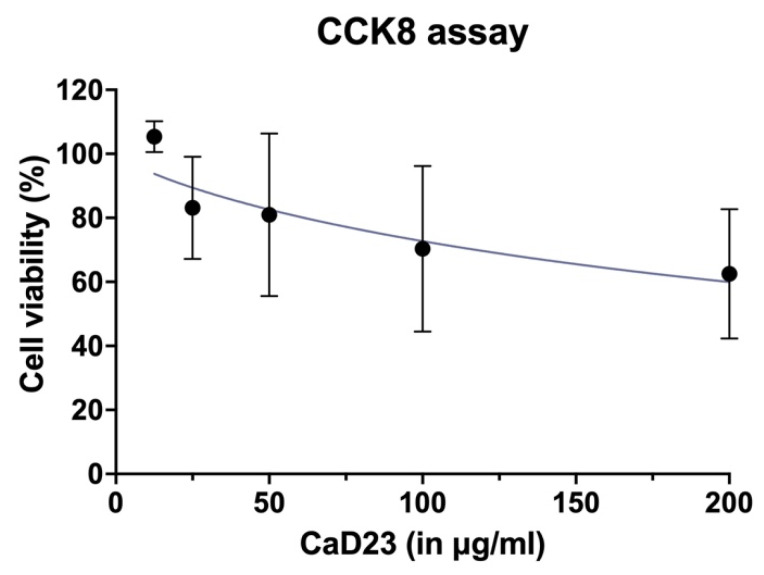
Cell viability CCK-8 assay of CaD23 in various concentrations against human corneal epithelial cells (HCE-S), presented as a dose–response curve (normalised, variable slope). The percentage cell viability is presented as mean ± standard deviation (depicted in error bars) of three independent biological experiments performed in technical duplicate. CCK-8 assay demonstrating a good safety profile of CaD23, with an IC_50_ of >200 μg/mL after 3 h of treatment. IC_50_ is defined as the treatment concentration that inhibits 50% of the cell viability.

The CCK-8 cell viability assay demonstrated a good safety profile of CaD23, with an IC_50_ of >200 µg/mL (or an estimated IC_50_ of 324.9 µg/mL based on a normalised dose–response curve [variable slope]; IC_50_ = treatment concentration that resulted in 50% inhibition of cell viability) (Figure 4). At a 200 µg/mL concentration of CaD23, the cell viability was 62.5 ± 16.5%. Based on the MIC of CaD23 against various strains of *S. aureus* (16–32 µg/mL), CaD23 exhibited a good therapeutic index (defined as IC_50_ divided by MIC) of around 10.2–20.3.

## 3. Discussion

Since the discovery of AMPs in the early 1980s, there has been a growing interest in the development of AMP as a novel antimicrobial therapy in view of their unique, rapid, and broad-spectrum antimicrobial activity against a wide range of organisms, including bacteria, fungi, and parasites, with a low risk of inducing AMR [25,26]. They are evolutionarily conserved molecular components of innate immunity that are found in all classes of life. They serve as an important first-line defence at many sites of the body, including the skin, respiratory, gastrointestinal and urinary tracts, ocular surface, and bloodstream [25,26]. In addition, other biological activities have demonstrated their significance, including antibiofilm, immunomodulatory, chemotactic, and wound healing properties, thereby positioning them as prospective therapeutic targets and alternatives to antibiotics [25,26]. Furthermore, they have been shown to synergise with currently available antibiotics, suggesting AMP–antibiotic combination therapy as a potential strategy to tackle AMR [33,34].

Previously, Ting et al. [30] showed that CaD23 could enhance the efficacy of amikacin and levofloxacin against several quality control, laboratory reference strains of *S. aureus* when CaD23–antibiotics are used in combination. In this study, we expanded the examination of the efficacy of CaD23 to ocular and systemic clinical isolates and its potential interaction with six different classes of antibiotics (with different mechanisms of action), including amikacin, levofloxacin, cefuroxime, fosfomycin, chloramphenicol, and vancomycin. This study provided several interesting and clinically meaningful findings that are beneficial for the management of ocular and systemic *S. aureus* infections, including bacterial keratitis.

Firstly, we showed that CaD23 has a good antimicrobial efficacy against a range of drug-susceptible and drug-resistant laboratory, ocular and systemic *S. aureus* (MIC = 16–32 μg/mL), highlighting that the efficacy of CaD23 is not influenced by the extent of resistance to conventional antibiotics (i.e., lack of cross-resistance between CaD23 and antibiotics). In addition, the efficacy of CaD23 remained largely stable in the presence of the physiological salt concentration, with a mostly 1-to-2-fold change in the MIC. Currently, topical antibiotics are the mainstay of treatment for bacterial keratitis, but the treatment options are limited. Recent studies have highlighted an increasing number of drug-resistant bacteria in BK, including MRSA, fluoroquinolone-resistant and multidrug-resistant *S. aureus* [14,18,19,20]. When compared to cases affected by drug-susceptible *S. aureus*, bacterial keratitis caused by drug-resistant *S. aureus* has worse clinical outcomes, including a higher rate of poor vision, longer healing time, and higher need for enucleation [19]. In addition, methicillin-resistant and multidrug-resistant *S. aureus* systemic infections are shown to be rising worldwide, posing a significant therapeutic challenge in current clinical practice [1,11,14,15]. In view of the lack of cross-resistance between CaD23 and various antibiotics, CaD23 may potentially serve as a useful therapy for treating drug-resistant *S. aureus*.

Secondly, as shown in a checkerboard assay, CaD23 enhances the antimicrobial efficacy of at least six different classes of conventional antibiotics when used in combination, serving as a useful therapeutic strategy for tackling drug-resistant *S. aureus.* The synergistic/strong additive effect of the combination treatment is likely attributed to the different mechanisms of action of CaD23 and antibiotics. In general, cationic AMPs possess amphipathic and hydrophobic properties that facilitate their initial binding to the anionic phospholipid bilayer of the bacterial cell membrane through electrostatic interaction, followed by the interaction with the lipid tail region of the membrane. This interaction leads to membrane permeabilisation, leakage of cytoplasmic contents and cell death, a mechanism distinct from those of conventional antibiotics [25]. Such a mechanism of action was similarly demonstrated in our previous study for CaD23 through a SYTOX Green uptake assay and molecular dynamics simulation studies [30]. In parallel with the previous study, the DiSC_3,5_ assay in this study showed that CaD23 (at 2× MIC or higher concentrations) was able to achieve significant inner membrane depolarisation within seconds of the treatment. Furthermore, these synergistic/additive interactions between AMPs and antibiotics have also been demonstrated by other research groups [34,35,36].

Amikacin, an aminoglycoside widely used in ophthalmology, exerts its antimicrobial activity by inhibiting the 30S ribosomal subunit [37], whereas levofloxacin, a commonly prescribed fluoroquinolone, eliminates bacteria through inhibition of bacterial topoisomerase IV (for Gram-positive bacteria) [38]. Cefuroxime, a β-lactam antibiotic, acts as a bactericidal agent by binding to penicillin-binding proteins (PBPs) located in the bacterial cell membrane, thereby inhibiting transpeptidase activity, disrupting septum formation, and ultimately leading to weakening and lysis of the bacterial cell wall [39]. Chloramphenicol acts as a bacteriostatic antibiotic, inhibiting bacterial protein synthesis by binding to the peptidyl transferase centre (PTC) on the 50S subunit of the bacterial ribosome [40]. On the other hand, fosfomycin is a bactericidal phosphoenolpyruvate (PEP) analogue that inhibits MurA, the enzyme that starts peptidoglycan synthesis in bacterial cell walls, causing bacterial cell wall failure and death [41]. Vancomycin, a glycopeptide antibiotic, kills Gram-positive bacteria via binding to the terminal D-alanyl-D-alanine portion of the growing cell wall, preventing cell wall cross-linking, and ultimately disrupting cell wall synthesis [42]. It is interesting to observe that the membrane permeabilising action of CaD23 could enhance the effect of all six different classes of antibiotics that inhibits either the cell wall synthesis or intracellular targets.

Thirdly, CaD23 was shown to have a significantly faster antimicrobial action (by 32-fold) than cefuroxime and levofloxacin, a commonly used antibiotic for treating Gram-positive keratitis (including *S. aureus*). Timely treatment and eradication of the infection are key to achieving a good outcome, including in both ocular and systemic infections. Therefore, the rapid antimicrobial action of CaD23 serves as a major advantage over conventional antibiotics. In addition, we did not observe any acceleration of the bacterial killing when the concentration of cefuroxime or levofloxacin was increased from 2× MIC to 32× MIC and 16× MIC, respectively, suggesting that the mechanism of action of cefuroxime and levofloxacin is more time-dependent instead of concentration-dependent. Encouragingly, we showed that CaD23 was able to expedite the antimicrobial action of cefuroxime from >8 h to 1 h, even when CaD23 was used at a sub-MIC concentration. While CaD23 did not expedite the antimicrobial action of levofloxacin, it was able to enhance the antimicrobial effect of levofloxacin by around 90% at an 8 h post-treatment time point. Therefore, CaD23–antibiotic combination treatment may serve as a valuable treatment strategy for tackling ocular and systemic *S. aureus* infections as it not only enhances the efficacy and efficiency of antibiotics but also reduces the peptide and antibiotic concentrations required for effective bacterial killing. This could also reduce the concentration-dependent toxicity of the peptide, which is usually a barrier for translating the therapy to the clinic [31]. The rapid antimicrobial action of CaD23 also helps reduce the risk of AMR evolution as compared to conventional antibiotics, which was observed in our previous study [29]. A previous large-scale evolutionary study similarly demonstrated that AMPs have a significantly lower risk of developing AMR when compared to conventional antibiotics [43].

For time–kill kinetics assays, we have focused on CaD23–cefuroxime and CaD23–levofloxacin combination therapy because these cefuroxime and levofloxacin are commonly used as the first-line treatment for treating Gram-positive bacterial infections, including *S. aureus* infections, in the setting of ocular infections and/or systemic infections [44,45]. In addition, based on checkerboard assays, CaD23–cefuroxime against MRSA-OS (an ocular clinical isolate) exhibited a synergistic effect (FICI = 0.344), whereas CaD23–levofloxacin against BH1CC (a systemic clinical isolate) exhibited an additive effect (FICI = 0.890). Therefore, in this study, we examined and compared the different combinations of CaD23–antibiotics with different interactive effects using time–kill kinetics assays to enable better interpretation and generalisability of the findings from the checkerboard assays. We have not performed detailed time–kill kinetics analyses for CaD23 with other antibiotics as the CaD23–amikacin combination has been examined in our previous study; fosfomycin and chloramphenicol are not commonly used as a first-line treatment; and vancomycin is often reserved as the last-line treatment for *S. aureus.*

In addition, we showed that CaD23 exhibited a good safety profile, with an estimated therapeutic index of 10–20. Generally, drugs are considered relatively safe if their therapeutic index exceeds the value of 10, whereas those with a therapeutic index of less than 3 require tighter monitoring and control [46]. The safety of CaD23 was further substantiated by the pre-clinical corneal wound healing model, which showed that CaD23 at 0.05% (or 500 µg/mL) did not show any in vivo toxicity [29].

The strengths of this work include examination of the efficacy of CaD23 against a range of laboratory, ocular and systemic *S. aureus* isolates and its interaction with six different classes of commonly used antibiotics. However, it would be beneficial to examine the CaD23–antibiotic interaction in more clinical strains of ocular and systemic *S. aureus* as well as Gram-negative organisms (e.g., *Pseudomonas aeruginosa*) to determine the generalisability of our study findings. While we showed that CaD23 exhibits good antimicrobial activities against a range of planktonic *S. aureus*, particularly when used in combination with conventional antibiotics, this study did not examine the efficacy of CaD23 against *S. aureus* biofilm, which is a major reason for the development of drug resistance in *S. aureus.* In addition, AMPs are known to be susceptible to proteolytic degradation and salt [47], which may affect the efficacy and stability of AMPs. Although the long-term stability (24 h) of CaD23 was not analysed in this study, the rapid antimicrobial action of CaD23 (achieving complete killing of bacteria within 15 min) suggests that the efficacy of CaD23 is unlikely to be affected by the long-term stability. We also showed that CaD23 remained relatively stable in the physiological salt concentration (with only a 1-to-2-fold change in MIC value). Nonetheless, future studies evaluating the antibiofilm efficacy and long-term stability (using high-performance liquid chromatography or mass spectrometry) would provide additional important information on CaD23 prior to clinical translation of the treatment. Another limitation of this study is the lack of in vivo validation of the observed synergistic effect between CaD23 and antibiotics in pre-clinical animal models. Future studies validating the effect of CaD23–antibiotic combination treatment in pre-clinical animal studies would be essential for advancing this therapeutic strategy to the clinic.

In conclusion, CaD23 represents a potentially valuable AMP for treating ocular and systemic drug-resistant *S. aureus* infections. Its ability to enhance the efficacy and efficiency of various classes of medically important antibiotics may serve as a potential solution to tackling AMR.

## 4. Materials and Methods

### 4.1. Study Design

This study was conducted using a range of in vitro microbiological experiments. All experiments were conducted in technical duplicates and repeated in two to three independent experiments. Relevant positive and negative/vehicle controls were used in all experiments described in this study. All continuous data were presented as mean ± standard deviation (SD).

### 4.2. Types of Microorganisms Used

A total of six methicillin-sensitive *S. aureus* (MSSA) and MRSA were used in this study. These included two quality control, laboratory reference strains of MSSA (ATCC SA29213 and SH1000), a quality control, laboratory reference strain of MRSA (ATCC MRSA43300), a community-acquired MRSA (USA300), and two MRSA clinical isolates (one isolated from the cornea/ocular surface of a patient with IK [MRSA-OS] and one isolated from the venous catheter of a patient with central venous catheter-related infection [BH1CC]). All laboratory reference strains were capable of causing ocular and systemic *S. aureus* infections.

### 4.3. Antibiotics and Peptide

Six medically important/last-line antibiotics (from six different classes), including second-generation cephalosporine (cefuroxime), third-generation fluoroquinolone (levofloxacin), aminoglycosides (amikacin), amphenicol (chloramphenicol), phosphonic acid (fosfomycin), and glycopeptide (vancomycin), were tested (Supplementary Table S1) [48]. All included antibiotics (except for fosfomycin) were commonly used antibiotics for ocular infections [44,49]. All antibiotics were purchased from Sigma-Aldrich (Merck Life Science UK Ltd., Dorset, UK). As per previous studies, the synthetic hybrid AMP, CaD23 (sequence: KRIVQRIKDWLRKLCKKW; molecular weight = 2398 g/mol), was commercially produced by Mimotopes (Mimotopes Pty. Ltd., Mulgrave VIC, Australia) via the traditional solid-phase Fmoc synthesis method. CaD23 was purified by reverse-phase high-performance liquid chromatography (RP-HPLC) to >95% purity and characterised by mass spectrometry.

### 4.4. In Vitro Antimicrobial Efficacy Assay

The in vitro efficacy of antibiotics and CaD23 was examined using the minimum inhibitory concentration (MIC) assay with the broth microdilution method approved by the Clinical and Laboratory Standards Institute (CLSI) [50]. Antibacterial susceptibility breakpoints were determined based on the CLSI and/or European Committee on Antimicrobial Susceptibility Testing (EUCAST) guideline (Supplementary Table S2).

Briefly, the bacteria were cultured on Luria–Bertani (LB) agar and incubated overnight at 37 °C for 18–24 h. Bacterial inoculums were then prepared using the direct colony suspension method according to the CLSI guideline [50]. Three to five bacterial colonies obtained from the agar plate were inoculated into an Eppendorf tube containing 1 mL of cation-adjusted Mueller–Hinton broth (MHB-2; Sigma-Aldrich, Merck Life Science UK Ltd., Dorset, UK). The bacterial suspension was adjusted to achieve a turbidity equivalent to 0.1 OD_600_, containing ∼1.0 × 10^8^ colony-forming unit (CFU)/mL of bacteria. The suspension was further diluted in 1:100 in MHB-2 to reach a final bacterial concentration of ~1 × 10^6^ CFU/mL. The treatments (antibiotics or CaD23) were prepared in 1:2 serial dilution in 96-well polypropylene (for CaD23 to reduce peptide adsorption to the well wall) or polystyrene microplates (for antibiotics), with a final treatment volume of 50 μL/well. An amount of 50 μL of 1 × 10^6^ CFU/mL bacterial suspension was added into each well in a 1:1 ratio (with a final bacterial concentration of 5 × 10^5^ CFU/mL). Growth control and sterility control were included in all assays. The MIC is defined as the lowest concentration of the treatment that inhibits the visible growth of bacteria after 21–24 h of incubation at 37 °C. To increase the robustness and reliability of the results, subjective visual interpretation of the bacterial growth (thence the MIC results) was also corroborated with the optical density read-outs using a plate reader (CLARIOstar, BMG Labtech, Aylesbury, UK). Furthermore, the MIC assay was performed in the presence of the physiological tear salt concentration (150 mM of NaCl), which is known to affect the efficacy of AMPs [31,51].

### 4.5. Checkerboard Assay

A checkerboard assay was performed to evaluate the potential synergistic interactions between antibiotics and CaD23 against all six strains of *S. aureus* using a previously established method [30]. Two 96-well plates were used for each experiment, including one polypropylene plate containing serial dilutions of CaD23 (Plate A, starting from 2–4× MIC), and one polystyrene plate containing serial dilutions of antibiotics (Plate B, starting from 4–8× MIC).

In Plate A, eight replicate horizontal rows of CaD23 were prepared and serially diluted (1:2 dilution) from column 1 to 7, with a final treatment volume of 25 µL/well. Column 8 was filled with 25 µL of vehicle only (i.e., sterile deionised water). In Plate B, eight replicate vertical rows of antibiotic were prepared and serially diluted (1:2 dilution) from row A to F, with a final treatment volume of 40 µL/well. Row G was filled with 40 µL of sterile deionised water only. Subsequently, 25 µL of treatment was transferred from each well of Plate B to the corresponding wells of Plate A to establish an equivalent 1:1 mixture of peptide and antibiotic. The bacterial suspension was then prepared and diluted to 1 ×10^6^ CFU/mL in fresh MHB-2. An amount of 50 µL of the diluted bacterial suspension was then added to each well, resulting in a final inoculum of 5 ×10^5^ CFU/mL in 100 µL total volume (1:1 of treatment and bacteria). Growth and sterility controls were included in all assay plates. After 21–24 h of incubation at 37 °C, the plates were assessed for growth.

**Figure 5:**
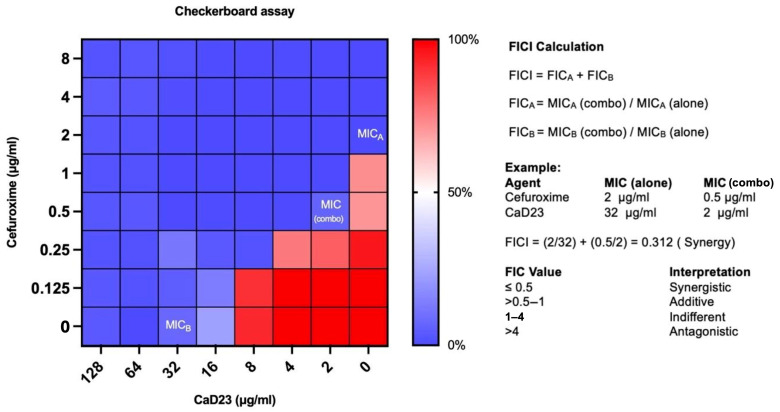
Checkerboard assay and fractional inhibitory concentration index (FICI) analysis of CaD23 and cefuroxime against MRSA-OS. Checkerboard assay heatmap illustrating the interaction between CaD23 (2–128 µg/mL) and cefuroxime (0.125–8 µg/mL) against MRSA-OS. Colour intensity indicates the percentage of bacterial growth after treatment (0–100%); red indicates strong growth (i.e., low inhibition) and blue indicates low growth (i.e., high inhibition). Calculation of the fractional inhibitory concentration index (FICI) for CaD23 and cefuroxime is demonstrated.

Peptide–antibiotic interaction was evaluated and interpreted based on the fractional inhibitory concentration index (FICI): synergistic (FICI ≤ 0.5), additive (FICI between >0.5 and 1.0), indifferent (FICI between >1.0 and ≤4), or antagonistic (FICI > 4) [30]. The FICI was calculated using the following formula: (MIC_CaD23(combined)_/MIC_CaD23(alone)_) + (MIC_antibiotic(combined)_/MIC_antibiotic(alone)_) (Figure 5) [30,52].

### 4.6. Time–Kill Kinetics Assay

A time–kill kinetics assay was performed to determine the time- and concentration-dependent antimicrobial action of CaD23 and antibiotics, namely cefuroxime and levofloxacin, both commonly used antibiotics for Gram-positive infections, including *S. aureus* infections. The bacterial suspension (1 × 10^6^ CFU/mL) was prepared using the same procedure method as described in the MIC assay. An amount of 100 μL of bacterial suspension was incubated with 100 μL of sterile deionised water in a 1:1 ratio and used as the growth control. Treatment groups were prepared in a 1:1 ratio (total volume of 200 μL, containing 100 μL of either peptide, antibiotic or both, and 100 μL of bacterial suspension). Treatment groups included CaD23 alone (2× MIC), cefuroxime alone (2× MIC), cefuroxime (8× MIC), and combined CaD23 (0.5× MIC) + cefuroxime (2× MIC). At 0 min, 15 min, 30 min, 1 h, 2 h, 4 h, 8 h, and 24 h, 10 μL of the treatment/bacteria mixture was removed from each well and was serially diluted (1:10) in sterile phosphate-buffered saline (PBS). The diluted suspension (20 μL) was subsequently removed and spread on LB agar in duplicate for bacterial enumeration after incubation for 18–21 h at 37 °C.

### 4.7. Cytoplasmic/Inner Membrane Depolarisation Assay (DiSC_3,5_)

A cytoplasmic membrane depolarisation assay was performed using a membrane potential sensitive probe, 3′,3′-dipropylthiadicarbocyanine (DiSC_3,5_) dye, to examine the mechanism of action of CaD23, based on our previous protocol with slight modifications [53]. The overnight culture of clinical isolate MRSA-OS was adjusted to OD_600_~0.01, then incubated for 1 h at room temperature in the dark with 10 μM of DiSC_3,5_ dye. CaD23 was prepared in a polypropylene treatment plate in 1:2 serial dilution (16× MIC to 4× MIC). After 1 h, the dye-loaded bacterial suspension was added to each well (100 μL per well) of a black plate, and the fluorescence intensity of each well was monitored using a plate reader (CLARIOstar, BMG Labtech, Aylesbury, UK), with excitation/emission wavelengths of 622/670 nm. Once the fluorescence signal was stable (after around 5–10 min), CaD23 treatment was then transferred to the black plate in a 1:1 ratio, with the final CaD23 concentrations ranging between 8×, 4×, and 2× MIC. Triton-X 1% was used as a positive control.

### 4.8. In Vitro Cell Viability and Cytotoxicity Assays

Cell viability and cytotoxicity of CaD23 peptides were determined against human corneal epithelial cells (HCE-S, an immortalised corneal epithelial cell line) using a cell counting kit-8 (CCK-8) assay (Sigma Aldrich, Merck Life Science UK Limited, Dorset, UK) as per the manufacturer’s guidelines. HCE-S cells were cultured and seeded into a 96-well plate at a density of 8000 cells/well and grew to 80–90% confluency over 48–72 h in phenol-free Dulbecco’s modified Eagle’s medium (DMEM) containing GlutaMAX^TM^-1 (4 mM), 1% penicillin–streptomycin, and 10% foetal bovine serum (Gibco, ThermoFisher Scientific, Abingdon, UK). The cells were then incubated with treatment (containing CaD23 and fresh media in 1:4 ratio) for 3 h. After 3 h of incubation, the treatment and media were removed and replaced with fresh media (90 μL of media + 10 μL of WST-8 dye), and the plate was incubated for 1–3 h before OD readings were obtained. Cells incubated with fresh media were used as growth controls, whereas wells containing only media and WST-8 dye were used as blank controls. The plate was read at 450 nm and 650 nm absorbance with the optical density read-outs using a plate reader (CLARIOstar, BMG Labtech, Aylesbury, UK), and the final OD of each well was calculated based on OD_450_ (dye signal + background noise) − OD_650_ (background noise). The following calculation formula was used to determine the cell viability (%):

Cell viability (%) = ((OD_treatment_ − OD_blank_)/(OD_control_ − OD_blank_)) × 100%

## Figures and Tables

**Table 1:** An antibiogram summarising the antibiotic susceptibility and resistance of two methicillin-sensitive *Staphylococcus aureus* (SH1000 and ATCC SA29213) and four methicillin-resistant *S. aureus* (BH1CC, ATCC MRSA43300, MRSA-OS, and USA300).

Bacteria	Minimum Inhibitory Concentration (MIC) Values (in µg/mL)						
	LFX	Amikacin	CPL	Fosfomycin	Vancomycin	Cefuroxime *	CaD23 **
SA29213	0.125	1	>128	8	0.25	1	32
SH1000	0.25	4	8	4	1	1	32
BH1CC	4	8	8	64	1	8	32
MRSA-OS	1	4	2	4	0.5	2	32
MRSA43300	0.125	8	16	16	0.25	1	32
USA300	8	4	8	16	0.5	>128	16

ATCC = American Type Culture Collection; CPL = chloramphenicol; LFX = levofloxacin; the antibiogram is encoded with red (resistance), yellow (intermediate), and green (susceptible) colours based on CLSI and/or EUCAST breakpoints. Intermediate susceptibility is considered as resistant in this study. * Cefuroxime susceptibility breakpoint against *Staphylococcus aureus* is inferred from cefoxitin breakpoint; ** there is no susceptibility breakpoint for CaD23 as this is a new drug that has not been used in humans.

**Table 2:** Summary of the minimum inhibitory concentration (MIC) of CaD23 against six strains of *Staphylococcus aureus* in the absence and presence of 150 mM NaCl. MIC values are presented in µg/mL. Data represent the result of two to three independent experiments.

Bacteria	MIC (in µg/mL)		Change in MIC
	CaD23 (No NaCl)	CaD23 (in 150 mM NaCl)	
USA300	16	16	No change
BH1CC	32	32	No change
MRSA43300	32	32	No change
SA29213	32	64	2-fold increase
MRSA-OS	32	128	4-fold increase
SH1000	32	Undetermined *	Undetermined

* Unable to determine as SH1000 was not able to grow well in the presence of 150 mM NaCl.

**Table 3:** Evaluation of the interactive effect of CaD23 and various antibiotics, including amikacin, levofloxacin, cefuroxime, chloramphenicol, fosfomycin, and vancomycin, using checkerboard assays. Experiments were conducted against two methicillin-sensitive *Staphylococcus aureus* (MSSA) and four methicillin-resistant *S. aureus* (MRSA).

Treatment	Bacteria	FICI *	Interpretation
CaD23 + Amikacin	SA29213	0.842 ± 0.308	Additive
	SH1000	0.521 ± 0.130	Additive
	USA300	0.625 ± 0.000	Additive
	BH1CC	1.113 ± 0.119	Indifferent
	MRSA43300	0.398 ± 0.099	Synergistic
	MRSA-OS	0.293 ± 0.185	Synergistic
CaD23 + Levofloxacin	SA29213	0.811 ± 0.262	Additive
	SH1000	0.813 ± 0.250	Additive
	USA300	0.625 ± 0.271	Additive
	BH1CC	0.890 ± 0.140	Additive
	MRSA43300	0.590 ± 0.045	Additive
	MRSA-OS	0.412 ± 0.346	Synergistic
CaD23 + Cefuroxime	MRSA-OS	0.344 ± 0.045	Synergistic
	BH1CC	0.766 ± 0.332	Additive
CaD23 + Chloramphenicol	MRSA-OS	0.583 ± 0.036	Additive
	BH1CC	0.813 ± 0.265	Additive
CaD23 + Fosfomycin	MRSA-OS	0.629 ± 0.402	Additive
	BH1CC	0.833 ± 0.567	Additive
CaD23 + Vancomycin	MRSA-OS	0.406 ± 0.190	Synergistic
	BH1CC	0.250 ± 0.00	Synergistic

FICI = fractional inhibitory concentration index; * FICI is calculated as (MIC_(CaD23(combined)_/MIC_(CaD23(alone))_) + (MIC_(antibiotic(combined))_/MIC_(antibiotic(alone))_). FICI is interpreted as: synergistic (≤0.5), additive (>0.5–1.0), indifferent (>1–4), and antagonist (>4). The results are based on two to three independent experiments. The results are presented as mean ± standard deviation.

## Data Availability

The original contributions presented in this study are included in the article/Supplementary Material. Further inquiries can be directed to the corresponding author.

## Supplementary Materials

The following supporting information can be downloaded at: https://www.mdpi.com/article/10.3390/ijms27125573/s1.

## References

[1] Ho C.S., Wong C.T.H., Aung T.T., Lakshminarayanan R., Mehta J.S., Rauz S., McNally A., Kintses B., Peacock S.J., de la Fuente-Nunez C. (2025). Antimicrobial resistance: A concise update. Lancet Microbe.

[2] O’Neill J. (2016). Tackling Drug-Resistant Infections Globally: Final Report and Recommendations.

[3] (2024). GBD 2021 Antimicrobial Resistance Collaborators. Global burden of bacterial antimicrobial resistance 1990–2021: A systematic analysis with forecasts to 2050. Lancet.

[4] Shi Z., Zhang J., Tian L., Xin L., Liang C., Ren X., Li M. (2023). A Comprehensive Overview of the Antibiotics Approved in the Last Two Decades: Retrospects and Prospects. Molecules.

[5] Hutchings M.I., Truman A.W., Wilkinson B. (2019). Antibiotics: Past, present and future. Curr. Opin. Microbiol..

[6] Howden B.P., Giulieri S.G., Lung T.W.F., Baines S.L., Sharkey L.K., Lee J.Y.H., Hachani A., Monk I.R., Stinear T.P. (2023). Staphylococcus aureus host interactions and adaptation. Nat. Rev. Microbiol..

[7] O’Callaghan R.J. (2018). The Pathogenesis of *Staphylococcus aureus* Eye Infections. Pathogens.

[8] Singh R.B., Singh Parmar U.P., Woreta F., Srikumaran D., Sharma N., Jhanji V. (2026). Global burden, risk factors, causative organisms and antibiotic susceptibility patterns in bacterial keratitis. Ocul. Surf..

[9] Ting D.S.J., Deshmukh R., Ting D.S.W., Ang M. (2023). Big data in corneal diseases and cataract: Current applications and future directions. Front. Big Data.

[10] Miller W.R., Arias C.A. (2024). ESKAPE pathogens: Antimicrobial resistance, epidemiology, clinical impact and therapeutics. Nat. Rev. Microbiol..

[11] An N., Hai L.H.L., Luong V.H., Vinh N.T.H., Hoa P.Q., Hung L., Son N.T., Hong L.T., Hung D.V., Kien H.T. (2024). Antimicrobial Resistance Patterns of Staphylococcus Aureus Isolated at a General Hospital in Vietnam Between 2014 and 2021. Infect. Drug Resist..

[12] Haaber J., Penadés J.R., Ingmer H. (2017). Transfer of Antibiotic Resistance in Staphylococcus aureus. Trends Microbiol..

[13] De Oliveira D.M.P., Forde B.M., Kidd T.J., Harris P.N.A., Schembri M.A., Beatson S.A., Paterson D.L., Walker M.J. (2020). Antimicrobial Resistance in ESKAPE Pathogens. Clin. Microbiol. Rev..

[14] Asbell P.A., Sanfilippo C.M., Sahm D.F., DeCory H.H. (2020). Trends in Antibiotic Resistance Among Ocular Microorganisms in the United States from 2009 to 2018. JAMA Ophthalmol..

[15] Vestergaard M., Frees D., Ingmer H. (2019). Antibiotic Resistance and the MRSA Problem. Microbiol. Spectr..

[16] Stapleton F. (2023). The epidemiology of infectious keratitis. Ocul. Surf..

[17] Green M., Apel A., Stapleton F. (2008). Risk factors and causative organisms in microbial keratitis. Cornea.

[18] Hsiao C.-H., Kang E.Y.-C., Yeh L.-K., Ma D.H.K., Chen H.-C., Hung K.-H., Huang Y.-C. (2022). Staphylococcus aureus Keratitis in Taiwan: Genotyping, Antibiotic Susceptibility, and Clinical Features. Int. J. Mol. Sci..

[19] Diacou R., Singh R.B., Romanowski E.G., Mandell J.B., Mammen A., Shanks R.M., Jhanji V. (2025). Moxifloxacin-resistant and moxifloxacin-susceptible Staphylococcus aureus Keratitis: Outcomes from a 10-year retrospective study. Ocul. Surf..

[20] Cabrera-Aguas M., Chidi-Egboka N., Kandel H., Watson S.L. (2024). Antimicrobial resistance in ocular infection: A review. Clin. Exp. Ophthalmol..

[21] Ting D.S.J., Bignardi G., Koerner R., Irion L.D., Johnson E., Morgan S.J., Ghosh S. (2019). Polymicrobial Keratitis with *Cryptococcus curvatus*, *Candida parapsilosis*, and *Stenotrophomonas maltophilia* After Penetrating Keratoplasty: A Rare Case Report with Literature Review. Eye Contact Lens.

[22] Khoo P., Cabrera-Aguas M.P., Nguyen V., Lahra M.M., Watson S.L. (2020). Microbial keratitis in Sydney, Australia: Risk factors, patient outcomes, and seasonal variation. Graefe’s Arch. Clin. Exp. Ophthalmol..

[23] Henry C.R., Flynn H.W., Miller D., Forster R.K., Alfonso E.C. (2012). Infectious keratitis progressing to endophthalmitis: A 15-year study of microbiology, associated factors, and clinical outcomes. Ophthalmology.

[24] Moussa G., Hodson J., Gooch N., Virdee J., Penaloza C., Kigozi J., Rauz S. (2021). Calculating the economic burden of presumed microbial keratitis admissions at a tertiary referral centre in the UK. Eye.

[25] Mookherjee N., Anderson M.A., Haagsman H.P., Davidson D.J. (2020). Antimicrobial host defence peptides: Functions and clinical potential. Nat. Rev. Drug Discov..

[26] Ting D.S.J., Mohammed I., Lakshminarayanan R., Beuerman R.W., Dua H.S. (2022). Host Defense Peptides at the Ocular Surface: Roles in Health and Major Diseases, and Therapeutic Potentials. Front. Med..

[27] Oliveira Júnior N.G., Souza C.M., Buccini D.F., Cardoso M.H., Franco O.L. (2025). Antimicrobial peptides: Structure, functions and translational applications. Nat. Rev. Microbiol..

[28] Hancock R.E.W., Alford M.A., Haney E.F. (2021). Antibiofilm activity of host defence peptides: Complexity provides opportunities. Nat. Rev. Microbiol..

[29] Ting D.S.J., Goh E.T.L., Mayandi V., Busoy J.M.F., Aung T.T., Periayah M.H., Nubile M., Mastropasqua L., Said D.G., Htoon H.M. (2021). Hybrid derivative of cathelicidin and human beta defensin-2 against Gram-positive bacteria: A novel approach for the treatment of bacterial keratitis. Sci. Rep..

[30] Ting D.S.J., Li J., Verma C.S., Goh E.T.L., Nubile M., Mastropasqua L., Said D.G., Beuerman R.W., Lakshminarayanan R., Mohammed I. (2021). Evaluation of Host Defense Peptide (CaD23)-Antibiotic Interaction and Mechanism of Action: Insights From Experimental and Molecular Dynamics Simulations Studies. Front. Pharmacol..

[31] Ting D.S.J., Beuerman R.W., Dua H.S., Lakshminarayanan R., Mohammed I. (2020). Strategies in Translating the Therapeutic Potentials of Host Defense Peptides. Front. Immunol..

[32] Ajish C., Yang S., Kumar S.D., Kim E.Y., Min H.J., Lee C.W., Shin S.-H., Shin S.Y. (2022). A novel hybrid peptide composed of LfcinB6 and KR-12-a4 with enhanced antimicrobial, anti-inflammatory and anti-biofilm activities. Sci. Rep..

[33] Taheri-Araghi S. (2024). Synergistic action of antimicrobial peptides and antibiotics: Current understanding and future directions. Front. Microbiol..

[34] Kampshoff F., Willcox M.D.P., Dutta D. (2019). A Pilot Study of the Synergy between Two Antimicrobial Peptides and Two Common Antibiotics. Antibiotics.

[35] Rajasekaran G., Kim E.Y., Shin S.Y. (2017). LL-37-derived membrane-active FK-13 analogs possessing cell selectivity, anti-biofilm activity and synergy with chloramphenicol and anti-inflammatory activity. Biochim. Biophys. Acta Biomembr..

[36] Wang G., Epand R.F., Mishra B., Lushnikova T., Thomas V.C., Bayles K.W., Epand R.M. (2012). Decoding the Functional Roles of Cationic Side Chains of the Major Antimicrobial Region of Human Cathelicidin LL-37. Antimicrob. Agents Chemother..

[37] Krause K.M., Serio A.W., Kane T.R., Connolly L.E. (2016). Aminoglycosides: An Overview. Cold Spring Harb. Perspect. Med..

[38] Fàbrega A., Madurga S., Giralt E., Vila J. (2009). Mechanism of action of and resistance to quinolones. Microb. Biotechnol..

[39] Lin X., Kück U. (2022). Cephalosporins as key lead generation beta-lactam antibiotics. Appl. Microbiol. Biotechnol..

[40] Ma X., Zhang L., Ren Y., Yun H., Cui H., Li Q., Guo Y., Gao S., Zhang F., Wang A. (2023). Molecular Mechanism of Chloramphenicol and Thiamphenicol Resistance Mediated by a Novel Oxidase, CmO, in *Sphingomonadaceae*. Appl. Environ. Microbiol..

[41] Silver L.L. (2017). Fosfomycin: Mechanism and Resistance. Cold Spring Harb. Perspect. Med..

[42] Stogios P.J., Savchenko A. (2020). Molecular mechanisms of vancomycin resistance. Protein Sci..

[43] Spohn R., Daruka L., Lázár V., Martins A., Vidovics F., Grézal G., Méhi O., Kintses B., Számel M., Jangir P.K. (2019). Integrated evolutionary analysis reveals antimicrobial peptides with limited resistance. Nat. Commun..

[44] Wespiser S., Koestel E., Fabacher T., Sauer A., Bourcier T. (2023). Practice patterns in the management of bacterial keratitis: A five-continent survey. Graefe’s Arch. Clin. Exp. Ophthalmol..

[45] Ting D.S.J., Cairns J., Gopal B.P., Ho C.S., Krstic L., Elsahn A., Lister M., Said D.G., Dua H.S. (2021). Risk Factors, Clinical Outcomes, and Prognostic Factors of Bacterial Keratitis: The Nottingham Infectious Keratitis Study. Front. Med..

[46] How Safe Is This Drug?. https://toxedfoundation.org/how-safe-is-this-drug/.

[47] Wendler J., Schroeder B.O., Ehmann D., Koeninger L., Mailänder-Sánchez D., Lemberg C., Wanner S., Schaller M., Stange E.F., Malek N.P. (2019). Proteolytic Degradation of reduced Human Beta Defensin 1 generates a Novel Antibiotic Octapeptide. Sci. Rep..

[48] WHO List of Medically Important Antimicrobials. https://cdn.who.int/media/docs/default-source/gcp/who-mia-list-2024-lv.pdf.

[49] Song A., Yang Y., Henein C., Bunce C., Qureshi R., Ting D.S.J. (2025). Topical antibiotics for treating bacterial keratitis: A network meta-analysis. Cochrane Database Syst. Rev..

[50] Clinical and Laboratory Standards Institute (2024). CLSI M07—Methods for Dilution Antimicrobial Susceptibility Tests for Bacteria That Grow Aerobically.

[51] Xu S., Tan P., Tang Q., Wang T., Ding Y., Fu H., Zhang Y., Zhou C., Song M., Tang Q. (2023). Enhancing the stability of antimicrobial peptides: From design strategies to applications. Chem. Eng. J..

[52] Odds F.C. (2003). Synergy, antagonism, and what the chequerboard puts between them. J. Antimicrob. Chemother..

[53] Ting D.S.J., Aung T.T., Mayandi V., Periayah M.H., Goh E.T.L., Muthu M., Barathi V.A., Mehta J.S., Tan D.T.H., Lakshminarayanan R. (2025). Biosynthetic ε-poly-L-lysine for the treatment of extensively- and pan-drug-resistant *Pseudomonas aeruginosa*. npj Antimicrob. Resist..

