# Supplementary material for: Evaluation of Antimicrobial Peptide–Antibiotic Combination Treatment for Tackling Ocular and Systemic Staphylococcus aureus Infections
Source: Int J Mol Sci. 2026 Jun 20;27(12):5573. doi: 10.3390/ijms27125573 (PMC13299516; doi:10.3390/ijms27125573)
Supplement: Supplementary file 1 [file ijms-27-05573-s001.zip › ijms-4364174-supplementary.pdf]

Supplementary Table S1. A summary of the mechanisms of action of the seven antibiotics used in this study.

Antibiotics	Mechanism of action
Amikacin	Inhibition of the 30S ribosomal subunit
Cefuroxime	Inhibition of bacterial cell wall synthesis
Chloramphenicol	Inhibits bacterial protein synthesis
Fosfomycin	Targeting peptidoglycan (cell wall) biosynthesis
Gentamicin	Inhibition of the 30S ribosomal subunit
Levofloxacin	Inhibition of bacterial topoisomerase IV
Vancomycin	Inhibition of bacterial cell wall synthesis

Supplementary Table S2. Antibiotic susceptibility breakpoints of various antibiotics against *Staphylococcus aureus* based on the CLSI and/or EUCAST guidelines.

Antibiotics	Susceptible (μg/ml)	Resistant (μg/ml)
Amikacin	≤ 8	> 16
Chloramphenicol	≤ 8	≥ 32
Cefuroxime*	≤4	≥ 4
Fosfomycin	≤ 32	≥ 32
Gentamicin	≤ 4	≥ 16
Levofloxacin	≤ 1	≥ 4
Vancomycin	≤ 2	≥ 16

*Susceptibility breakpoint of cefuroxime is inferred from the cefoxitin susceptibility.
